# Imaging diseases of the aorta by MRI: a cost-effectiveness analysis of contrast-enhanced studies compared to non-contrast enhanced angiographic studies

**DOI:** 10.1186/1532-429X-14-S1-P45

**Published:** 2012-02-01

**Authors:** Emer Sonnex, Melissa L Grzeszczak, Richard Coulden

**Affiliations:** 1 Diagnostic Imaging, University of Alberta Hospitals, Edmonton, AB, Canada

## Summary

Non-contrast-enhanced MR angiography (MRA) of the aorta is a robust alternative to using contrast-enhanced (CE) angiography in the imaging of thoracic aortic diseases. What is the cost impact of not using intravenous contrast routinely in thoracic aorta MRA?

## Background

Free-breathing, ECG-triggered, navigator-gated 3D segmented steady state free precession (SSFP) MRA is a promising MR technique for imaging the thoracic aorta. We wanted to establish the economic impact of using non-contrast enhanced angiography (non-CEMRA) in our unit compared to the same time period last year when only CEMRA was used.

## Methods

Over a 3 month period, 22% of referrals to our cardiac MRI unit (Siemens Aera©, Erlangen, Germany) were for diseases of the aorta. These patients were divided into 5 subsets: 1) familial screening (Marfans, Turners, etc), 2) dilatation, 3) follow-up aortic dissection, 4) congenital aortic and valve disease & 5) vasculitis. 80 data sets for patients referred for evaluation of their aorta were examined. These were compared with 80 data sets in patients with aortic disease imaged in a similar period when only CEMRA was available. Clinical evaluation included recording the patients' serum creatinine, correlation with aortic measurements from previous imaging studies and subjective assessment of image quality. Cost analysis included costs of contrast agent, power injector sets and routine consumable costs associated with intravenous access.

## Results

The unit price for consumables for thoracic aortic CEMRA was estimated at $140CAD*/patient ($110CAD for Gadolinium and $30CAD for other disposables). Vasculitis patients (group 5) were excluded from both data sets as intravenous contrast was needed for delayed enhancement imaging. No patient was excluded from CE-angiography due to a high serum creatinine (eGFR in all patients ≥ 30mls/min). No patient undergoing non-CEMRA required an additional CEMRA for a non-diagnostic study. We examined 75 data sets in the 'pre-navigator' group and 72 in the 'post-navigator' group. In the pre-navigator group, 50 patients (67%) underwent CEMRA as part of their examination: 9/12 (75%) group 1, 12/28 (43%) group 2, 2/3 (67%) group 3 and 27/32 (84%) group 4. In the post-navigator group, 14 patients (19%) underwent CEMRA: 5/18 (28%) group 1 (268% reduction), 6/31 (19%) group 2 (266% reduction), 1/5 (20%) group 3 (335% reduction) and 5/18 (28%) group 4 (300% reduction). Costs associated directly with CEMRA in the pre-navigator group were $7000CAD (50 x $140CAD) and in the post-navigator group were $1960CAD (14 x $140CAD), a saving of $5040.

*CAD = Canadian dollars.

## Conclusions

We conclude that using a free-breathing, ECG-triggered, navigator-gated 3D segmented steady state free precession (SSFP) sequence is a cost-effective way of imaging disease of the aorta without compromising imaging quality.

## Funding

Not applicable.

**Table 1:** Number of CEMRA performed

	Group 1 familial screening	Group 2 dilatation	Group 3 dissection	Group 4 aortic congenital disease	Group 5 vasculitis
Pre-navigator	9/12 (75%)	12/28 (43%)	2/3 (67%)	27/32 (84%)	5/5 (100%)
Post-navigator	5/18 (28%)	6/31 (19%)	1/5 (20%)	5/18 (28%)	8/8 (100%)
% reduction	268%	226%	335%	300%	not applicable

